# Lack of Support for the Genes by Early Environment Interaction Hypothesis in the Pathogenesis of Schizophrenia

**DOI:** 10.1093/schbul/sbab052

**Published:** 2021-05-14

**Authors:** Evangelos Vassos, Jiaqi Kou, Sarah Tosato, Jessye Maxwell, Charlotte A Dennison, Sophie E Legge, James T R Walters, Michael J Owen, Michael C O’Donovan, Gerome Breen, Cathryn M Lewis, Patrick F Sullivan, Christina Hultman, Mirella Ruggeri, Muriel Walshe, Elvira Bramon, Sarah E Bergen, Robin M Murray

**Affiliations:** 1 Social, Genetic and Developmental Psychiatry Centre, Institute of Psychiatry, Psychology & Neuroscience, King’s College London, London, UK; 2 NIHR Maudsley Biomedical Research Centre, South London and Maudsley NHS Trust, London, UK; 3 Department of Medical Epidemiology and Biostatistics, Karolinska Institutet, Stockholm, Sweden; 4 Section of Psychiatry, Department of Neuroscience, Biomedicine and Movement Sciences, University of Verona, Verona, Italy; 5 Medical Research Council Centre for Neuropsychiatric Genetics and Genomics, School of Medicine, Cardiff University, Cardiff, UK; 6 Center for Psychiatric Genomics, Department of Genetics and Psychiatry, University of North Carolina, Chapel Hill, NC; 7 Division of Psychiatry, University College London, London, UK; 8 Department of Psychosis Studies, Institute of Psychiatry, Psychology & Neuroscience, King’s College London, London, UK

**Keywords:** psychosis, polygenic risk score, obstetric complications, early life events, gene environment interaction, placenta biology

## Abstract

Ursini et al reported recently that the liability of schizophrenia explained by a polygenic risk score (PRS) derived from the variants most associated with schizophrenia was increased 5-fold in individuals who experienced complications during pregnancy or birth. Follow-up gene expression analysis showed that the genes mapping to the most associated genetic variants are highly expressed in placental tissues. If confirmed, these findings will have major implications in our understanding of the joint effect of genes and environment in the pathogenesis of schizophrenia. We examined the interplay between PRS and obstetric complications (OCs) in 5 independent samples (effective *N* = 2110). OCs were assessed with the full or modified Lewis-Murray scale, or with birth weight < 2.5 kg as a proxy. In a large cohort we tested whether the pathways from placenta-relevant variants in the original report were associated with case-control status. Unlike in the original study, we did not find significant effect of PRS on the presence of OCs in cases, nor a substantial difference in the association of PRS with case-control status in samples stratified by the presence of OCs. Furthermore, none of the PRS by OCs interactions were significant, nor were any of the biological pathways, examined in the Swedish cohort. Our study could not support the hypothesis of a mediating effect of placenta biology in the pathway from genes to schizophrenia. Methodology differences, in particular the different scales measuring OCs, as well as power constraints for interaction analyses in both studies, may explain this discrepancy.

## Introduction

Obstetric complications have been consistently associated with schizophrenia and are considered nongenetic risk factors for the disease.^1^ They include a variety of complications of pregnancy (eg, infections, diabetes, preeclampsia, bleeding), fetal growth (low birth weight, prematurity), and delivery (prolonged labor, fetal hypoxia, or asphyxia). Despite the relatively small effect sizes with odds ratios of less than 2, the association appears robust, with numerous replications summarised in a recent meta-analysis.^2^ These observations were important in supporting the theoretical framework of the “neurodevelopmental hypothesis” of schizophrenia, first presented in 1987,^3,4^ which suggests that the combination of genetic predisposition coupled with pre- or perinatal events affects brain development and increases vulnerability to schizophrenia later in life.

Genome-wide association studies (GWAS) have identified many risk variants robustly associated with schizophrenia.^5,6^ The total genetic effect of common variants is usually summarised in polygenic risk scores (PRSs), which have proved powerful predictors of disease.^7^ However, there is still distance to be covered in understanding the pathways linking genetic variation with disease development. Furthermore, because PRS is a composite measure, it tells us nothing about the interactions of individual genes with the environment. One idea gaining traction these days is that traditional distinctions between genetic and environmental risk are blurred. It has been suggested that GWAS of disease outcomes have identified genetic variants that capture the effect of modifiable risk factors as well as direct genetic effects.^8^

In this context, Ursini and colleagues^9^ recently reported that the intrauterine and perinatal environment modulates the association of schizophrenia with genomic risk, by examining the interaction between PRS for schizophrenia with the presence of obstetric complications, which they termed early-life complications (ELCs). They reported that the association of PRS with schizophrenia is 5-fold stronger in patients with ELCs compared to patients without ELCs and that the finding replicated in 2 independent samples. Follow-up gene expression and pathway analyses showed that the genes mapping to the most associated genetic variants with schizophrenia are highly expressed in placental tissues and have differential expression in placentae from complicated pregnancies.

Ursini et al thus proposed a mechanism of action for the genetic variants most associated with schizophrenia leading to altered neurodevelopment through modulation of placental physiology and function. This report suggests that some of the top risk variants for schizophrenia may influence the health of the placenta and render a foetus less resilient to early hazards. If confirmed, these findings would have major implications for our understanding of the joint effect of genes and environment in the pathogenesis of schizophrenia.

Examining the data of Ursini et al,^9^ it is striking that the interaction with ELCs was found only in PRS based on a relatively small number of variants at or near genome-wide significance (*P*_T_ < 5 × 10^−8^ and *P*_T_ < 10^–6^), at thresholds which are usually less predictive than PRS built on a larger set of variants.^5^ Accepting that schizophrenia is a polygenic disorder and its aetiopathological mechanisms are likely to be diverse,^10^ one explanation is that the more strongly associated polymorphisms act via altered placental transcriptional regulation, while variants associated at lesser thresholds of significance act through different pathophysiological pathways. To confirm this hypothesis, we tested whether genetic risk from the most associated loci with schizophrenia is modulated through pre- or perinatal events in independent samples with genetic and obstetric complications (OCs) data.

## Methods

### Sample Description

Our study included 3 clinical case-control samples: (1) a subsample of the Swedish Schizophrenia Study (Sweden)^11^ comprising 310 schizophrenia cases and 237 controls with data on obstetric complications; (2) a sample from London (Maudsley Family Study; MFS)^12^ with 60 cases with psychotic disorder, 47 unaffected first degree relatives of cases and 17 controls; and (3) a sample of first-episode psychosis from Italy (Verona)^13^ comprising 141 cases and 89 controls. In addition, we analyzed (4) a population sample from the UK Biobank (UKB),^14^ where we identified 326 cases with schizophrenia based on self-reporting or Hospital Episode Statistics and 220,582 unaffected controls; and (5) a case-only sample (Cardiff)^15^ of 804 individuals with schizophrenia or schizoaffective disorder, to test whether PRS predicts OCs in cases. Detailed descriptions of the samples are reported in the supplementary material. Effective sample sizes for the case-control samples were estimated using the formula *N*_*eff*_ = 4/(1/*N*_cases_ + 1/*N*_controls_).

### Obstetric Complication Measures

OCs were assessed with the Lewis-Murray scale,^16^ a 15-item scale consisting of complications that happened during the ante- and intrapartum period. This is a widely used and validated scale.^2,17^ Individuals were scored as “positive” if they had at least one definite complication, and OCs were used as a binary outcome. In the UKB, in the absence of detailed data of pre- or perinatal events, we used birth weight < 2.5 kg as a proxy for OCs, taking into account the effect size and the proportion of the population exposed to the risk, and in line with previous evidence that low birth weight is among the most significant and consistent OCs associated with risk for schizophrenia.^2,18^

### Genetic Data and PRS Analysis

Genotyping, quality-control, and imputation methods for each of the included samples have been published previously.^11,19–21^ In brief, genotyped SNPs were removed if they had missingness > 0.02, minor allele frequency (MAF) < 0.01, or Hardy-Weinberg equilibrium *P*-value < 10^−06^ (10^−08^ in UKB). Participants who were related based on identity-by-descent values, with mismatching reported and genotyped sex, or with call rates < 98%, were removed from the datasets. PCA using LD pruned variants was performed in PLINK.^22^ Only genotypically confirmed European ancestry individuals based on the first 2 principal components were included in the analyses. PRS with the clumping and thresholding method^23^ for the 4 case-control samples were built using the published Psychiatric Genomics Consortium (PGC) schizophrenia GWAS^5^ and for the case-only sample from Cardiff the meta-analysis with the CLOZUK discovery samples.^21^ For samples included in the discovery GWAS, we used leave-one-out training datasets.

All analyses were repeated with PRS formed from SNPs associated with schizophrenia at the 2 levels of significance, *P*_T_ < 5 × 10^−8^ and P_T_ < 10^−6^, that showed interaction with ELCs in the Ursini et al^9^ study. We also repeated the analyses at *P*_T_ < 0.1, which consistently showed high association with case-control status in our samples. All PRS included in the analyses were adjusted for population stratification by using the first 10 principal components (PC) as covariates, with the exception of MFS (our smallest sample) where the model did not converge and we restricted our covariates to 4 PCs. Analyses were performed with logistic regression models for: (1) association of PRS with OCs in total samples and stratified by case or control status, (2) association of PRS with case-control status in the total samples and stratified by the presence or absence of OCs, and (3) PRS by OCs interaction on case-control status including as covariates all possible PC by PRS and PC by OCs interactions.^24^ Effect sizes from our different samples were pooled together with fixed effects meta-analyses due to low heterogeneity as measured with the *I*^2^ statistic.^25^

### Pathway Analysis

Ursini et al^9^ reported that the SNPs driving the PRS-OC interaction lie in pathways enriched for “placental” genes (ie, genes highly expressed in placentae and differentially expressed in placentae from complicated pregnancies). This was conducted by using the subset of SNPs from PRS at *P*_T_ < 5 × 10^−8^ and *P*_T_ < 10^−6^ deemed relevant to placenta biology and conducting a pathway analysis that yielded 7 and 67 significant pathways, respectively. To examine whether these gene-sets are associated with schizophrenia risk, we conducted pathway analyses using these placenta-relevant SNPs indexed in the summary statistics from a GWAS of the full Swedish Schizophrenia Study with a total sample of 11 244 subjects (5001 cases and 6243 controls) and 9 871 789 imputed SNPs.^11^

Pathway analysis using MAGMA v1.09^26^ consists of 3 steps: first, mapping SNPs onto genes by an annotation step; second, computing gene level *P*-values by a gene analysis step; and third, computing pathway level *P*-values by a gene-set analysis step. Gene locations for the annotation step used NCBI Build 37 / UCSC hg19 to ensure that the same human genome build as the SNP locations in the previous GWAS study were used. A competitive test was performed, which tests the hypothesis that the statistics of genes within a pathway are significantly different from genes outside the pathway. A Bonferroni correction was applied, resulting in adjusted significance thresholds of *P* < .007 for the first and *P* < .0007 for the second PRS threshold for the 7 and 67 pathways tested, respectively.

## Results

The presence of OCs was not associated with case-control status, similar to the Ursini et al discovery sample, possibly reflecting power limitations in both studies (total effective sample size estimated 1644 in Ursini et al and 2110 in our study). Following similar methodology, we examined (1) whether PRS at *P*_T_ < 5 × 10^−8^ and *P*_T_ < 10^−6^ is associated with the presence of OCs preferentially in cases, (2) whether the association between PRS and case-control status is stronger in the presence of OCs and (3) the interaction between PRS and OCs in predicting case-control status. Reported *P*-vales are 2-sided.

In none of the 5 samples we examined, was PRS at *P*_T_ < 5 × 10^−8^ associated with the presence of OCs either in total samples or specifically in cases. We only observed a positive association of PRS at *P*_T_ < 10^−6^ with history of OCs in the total Swedish sample (*P* = .03) and in MFS cases (*P* = .04), which would not survive any correction for multiple testing (table 1). Our meta-analysis results were not significant (*P* = .60 at *P*_T_ < 5 × 10^−8^ and *P* = .31 PRS at *P*_T_ < 10^−6^; supplementary figure 1).

**Table 1.** Associations Between OCs and PRS at *P*_T_ < 5 × 10^−8^ and *P*_T_ < 10^−6^

			*P*_*T*_ < 5 × 10^−8^		*P*_*T*_ < 10^−6^	
Sample		*N*	*exp(B)*	*P*	*exp(B)*	*P*
Sweden	All	547	1.15	.16	1.23	**.03**
	Controls	237	1.26	.12	1.17	.29
	Cases	310	1.04	.75	1.28	.05
Verona	All	230	0.99	.96	0.90	.49
	Controls	89	0.94	.78	0.86	.54
	Cases	141	1.07	.76	0.97	.89
MFS	All	123	0.98	.91	1.22	.36
	Controls	17	0.43	.19	0.48	.28
	Relatives	47	0.30	.05	0.27	**.04**
	Cases	59	1.43	.26	2.04	**.04**
UKB	All	220 908	1.003	.71	1.004	.55
	Controls	220 582	1.003	.71	1.004	.55
	Cases	326	0.97	.86	1.02	.91
Cardiff	Cases	804	1.01	.89	0.95	.57

*Note:* MFS, Maudsley Family Study; OC, obstetric complication; PRS, polygenic risk score; UKB, UK Biobank. Association between PRS and history of OCs in the total sample (All), controls, cases (and relatives in the MFS) separately. OCs, obstetric complications; PRS, schizophrenia polygenic risk score. In bold significant results at *P* < .05

Associations of PRS at *P*_T_ < 5 × 10^−8^ and *P*_T_ < 10^−6^ with case-control status were significant only in the largest samples (Swedish and UKB). Stratification by the presence of OCs did not appear to have a substantial or consistent effect. For example, PRS at *P*_T_ < 10^−6^ in the Swedish sample appeared to have a stronger effect in the presence of OCs (odds ratio 1.62 vs 1.57 in the absence of OCs), but when examining PRS at *P*_T_ < 5 × 10^−8^ at the same sample we notice the opposite effect (stronger effect of PRS in the absence, nonsignificant in the presence of OC) (table 2). Subgroup meta-analysis stratified by the presence of OCs did not show substantial heterogeneity or difference in effect sizes between the group means (supplementary figure 2). Furthermore, none of the PRS by OCs interactions we tested were significant (table 3).

**Table 2.** Associations Between PRS and Case-Control Status Stratified by the Presence of OCs

			*P*_*T*_ < 5 × 10^−8^			*P*_*T*_ < 10^−6^		
Sample		*N*	*exp(B)*	*R* ^2^	*P*	*exp(B)*	*R* ^2^	*P*
Sweden	All	547	1.48	0.030	**2.2E-04**	1.59	0.053	**1.9E-06**
	OCs−	368	1.49	0.040	**7.8E-04**	1.57	0.050	**1.1E-04**
	OCs+	179	1.18	0.006	.34	1.62	0.046	**.01**
Verona	All^a^	298	1.03	0.000	.85	1.12	0.003	.45
	OCs−	154	0.97	0.000	.86	1.03	0.000	.87
	OCs+	76	1.17	0.004	.57	1.23	0.007	.45
MFS	All^a^	170	1.13	0.002	.71	1.22	0.006	.6
	OCs−	48	0.82	0.005	.65	0.86	0.003	.73
	OCs+	28	1.95	0.061	.26	3.08	0.112	.15
UKB	All	220 908	1.26	0.003	**2.8E-04**	1.33	0.005	**8.0E-06**
	OCs−	198 739	1.27	0.003	**3.6E-04**	1.33	0.004	**2.2E-05**
	OCs+	22 169	1.16	0.001	.39	1.30	0.004	.154

*Note:* MFS, Maudsley Family Study; OC, obstetric complication; PRS, polygenic risk score; UKB, UK Biobank. Association between PRS and case-control status in the total sample (All), the subsample without OCs history (OCs−), and the subsample with OCs history (OCs+). *R*^2^, the Nagelkerke *R*^2^. Significant results in bold.

^a^In Verona and MFS, the total sample (All) is higher than the sum of the OCs− and OCs+ as individuals with missing OC data were included.

**Table 3.** Interaction Between PRS and OCs on Case-Control Status

		PRS at *P*_*T*_*<* 5 × 10^−8^		PRS at *P*_*T*_*<* 10^−6^	
Sample	*N*_*eff*_	*exp(B)*	*P*	*exp(B)*	*P*
Sweden	537	0.73	.18	0.99	.96
Verona	218	1.24	.59	1.25	.57
MFS	53	3.20	.22	4.20	.18
UKB	1302	0.92	.66	0.98	.90

*Note:* MFS, Maudsley Family Study; OC, obstetric complication; PRS, polygenic risk score; UKB, UK Biobank. Coefficients and *P*-values from the interaction term PRS*OC in the logistic regression model. *N*_*eff*_, Effective sample sizes.

Given that the main effect of PRS on affected status increases when relaxing the threshold as expected from previous work with current sized GWAS training dataset,^5^ we repeated the analyses at *P*_T_ < .1. At this threshold, PRS was associated with case-control status in all the samples with odds ratios varying depending on sample definition and sample size (range 1.62–2.87). With the exception of MFS, we did not observe any preferential association between PRS and OCs in cases or any consistent effect of OC stratification on the association between PRS and case-control status (supplementary table 1).

The pathways enriched for placental genes at both threshold levels (*P*_T_ < 5 × 10^−8^ and *P*_T_ < 10^−6^) as reported in the study by Ursini et al were tested for association with case-control status in the total Swedish cohort. No significant associations were found even at a nominal level of significance (supplementary tables 2 and 3).

## Discussion

We failed to confirm the findings by Ursini et al^9^ both in terms of modulation of the effect of PRS by OCs and association of PRS for schizophrenia with history of OCs in cases. Despite the consistent findings across samples in the original report, disappointingly we did not find similar associations or even trends in any of the 5 samples we tested.

Methodology differences, in particular the different scales measuring OCs, for interaction analyses in both studies, may explain this discrepancy. The study by Ursini et al measured OCs using the McNeil–Sjöström scale,^27^ while we used the Lewis-Murray scale^16^ in 3 of the samples, an adaptation in the Cardiff sample and only birth weight in the UKB. The 2 scales have differences: The Lewis-Murray scale has been the more frequently used, and an individual patient meta-analysis of 12 studies showed that it distinguished significantly between patients with schizophrenia and controls.^28^ However, it is less detailed than the McNeil–Sjöström scale. A study by McNeil et al^29^ comparing the scales, showed that both scales discriminated OC histories between singletons with schizophrenia and controls, but the McNeil–Sjöström scale functioned best in discriminating OC histories between schizophrenic twins and control twins. However, it is unclear whether the differences between the 2 scales can explain the paucity of effect in our study.

From the latest meta-analysis,^2^ we notice that most of the strongly associated OCs (eg, maternal infections, premature rupture of membrane, premature birth, low birth weight, and congenital malformation) are captured by the Lewis-Murray scale, with few important exceptions (Herpes simplex type 2, famine, maternal hypertension, asphyxia). The pooled effect from 9 studies of definite obstetric complications as specified in the Lewis-Murray scale was a significant predictor of case-control status (OR = 1.83, *P*-value = .0042). This suggests that the Lewis-Murray scale is efficient in capturing relevant OCs, and differences between the findings of our study and that of Ursini et al is possibly due to the additional items covered only in the McNeil–Sjöström scale. Further comparing the 2 studies, we notice that our samples had more consistent prevalence of OCs, between 25% and 36% for the full Lewis-Murray scale and 10% for low birth weight in the UKB. More variability in OCs was observed in the Ursini et al study, with the range of ELCs prevalence between 20% and 66%, reflecting either more variability in the sample selection or in the ratings of the McNeil–Sjöström scale.

Other considerations are the differences in the sample characteristics between the 2 studies and power constraints. Two of our samples (MFS, Verona) included a broader definition of psychosis, not restricted to schizophrenia and schizoaffective disorder. However, given the evidence that OCs is a risk factor across non-organic psychoses^2^ and the high genetic correlation of schizophrenia with bipolar disorder,^30,31^ it is unlikely that this has an important impact on the findings. In terms of the power to detect gene by environment interaction, both studies utilized a variety of samples with genetic and OC data. It is noteworthy that, although the initial findings were replicated in 2 additional case-control samples in the Ursini et al study, we failed to confirm the association, despite similar total effective sample size (1644 in Ursini et al and 2110 in our study). To further test the power of our samples for PRS analyses, we repeated the analyses with a more powerful PRS at *P*_T_ < .1 and we found significant main effects in all samples, as expected, but no interaction effects.

Our failure to confirm the interaction is not surprising, given the vanishingly rare evidence in the literature that average genetic risks for chronic diseases vary substantially according to environmental exposures.^32^ Studies of gene-environment interaction suffer from low power to detect these effects,^33^ while the violation of underlying model assumptions increases the likelihood of spurious findings.^34^ For studies of interaction between polygenic scores and environment, methodological and conceptual challenges, including the choice of the environment and outcome variables, measurement error, and sample selection processes, can result in misleading outcomes.^35^

Given the above limitations in the PRS by OCs interaction analyses, to further examine the hypothesis that placenta biology is a significant mediator of the genetic risk and the development of schizophrenia, we tested whether the significant pathways identified by Ursini et al were enriched in schizophrenia cases. Our hypothesis was that if these pathways are important in the pathogenesis of schizophrenia, they should be associated with case-control status in a large sample of 5001 schizophrenia cases and 6243 controls. However, our null pathway analysis results call into question the relevance of these pathways.

In summary, although we cannot categorically refute the original findings due to differences in the study design, we would like to add a note of caution that early environment modulation of the effect of PRS may be population specific or related to specific early life events not captured by the scale we used. Our study, in line with empirical data to this point in psychiatric and behavioral genetics, not having identified many replicable examples for gene by environment interaction, highlights the complexity of the field. Further research with larger, well-phenotyped samples is advisable to examine the interaction of genetic liability with obstetric complications and similarly with other environmental risks with the aim of unravelling specific aetiopathogenic mechanisms of schizophrenia.

## Funding

This study represents independent research funded by the National Institute for Health Research (NIHR) Biomedical Research Centre at South London and Maudsley NHS Foundation Trust and King’s College London. The views expressed are those of the authors and not necessarily those of the NHS, the NIHR or the Department of Health and Social Care. Part of this research has been conducted using the UK Biobank Resource under Application Number 18177 (PI Cathryn Lewis). The work at Cardiff University was supported by Medical Research Council Centre Grant no. MR/L010305/1 and Program Grant no. G0800509. R.M. is partly supported by a grant from David Posnett.

## Conflicts of Interest

The authors have declared that there are no conflicts of interest in relation to the subject of this study. M.C.O.D., J.T.R.W. and M.J.O. have received a collaborative research grant from Takeda Pharmaceuticals. Takeda played no part in the conception, design, implementation, or interpretation of this study.

## Supplementary Material

sbab052_suppl_Supplementary_Material
