# Supplementary material for: Lack of Support for the Genes by Early Environment Interaction Hypothesis in the Pathogenesis of Schizophrenia
Source: Schizophr Bull. 2021 May 14;48(1):20–6. doi: 10.1093/schbul/sbab052 (PMC8781344; doi:10.1093/schbul/sbab052)
Supplement: sbab052_suppl_Supplementary_Material [file sbab052_suppl_supplementary_material.docx]

**Lack of support for the genes by early environment interaction hypothesis in the pathogenesis of schizophrenia**

**Supplementary Material**

**Sample description**

**1. Sweden**

Samples of this case-control study were obtained from the Swedish Schizophrenia Study,^1^ which comprised 5,351 cases and 6,509 controls. Cases were identified via the Hospital Discharge Register, which gathers all public and private impatient hospitalizations. The register was complete from 1987, and contains diagnoses coded using the International Classification of Disease (ICD) discharge diagnoses made by attending physicians for each hospitalization. Inclusion criteria for cases were ≥18 years of age, having Nordic ancestry and at least two hospitalizations for schizophrenia to enhance validity. Controls were randomly selected from the Swedish population registers. Inclusion criteria for controls were ≥18 years of age, both parents born in Scandinavia and have never received a discharge diagnosis of schizophrenia or bipolar disorder diagnosis. The study participants have been genotyped in 6 waves (denoted Sw1-Sw6) using the Affymetrix 5.0 SNP array (Sw1), Affymetrix 6.0 (Sw2-Sw4) and Illumina OmniExpress (Sw5 and Sw6). Genotyping, quality-control, and imputation methods have been published previously. These samples have been linked to several of the Swedish National Registers (including the Hospital Discharge Register and the Medical Birth Register), most recently in 2014.

Data on ELCs were obtained from the Swedish Medical Birth Register, which was founded in 1973 and included data on almost all deliveries in Sweden. Many of the participants in the Swedish Schizophrenia Study were born before the establishment of the Medical Birth Register, but 547 samples in this study were able to be linked to this register. Thus, the total sample for the current study consisted of 547 Swedish subjects (310 cases and 247 controls). The ELCs were defined using the Lewis-Murray scale,^2^ a 15-item scale consisting of complications which happened during prenatal, delivery and neonatal period. Each item in the Lewis-Murray scale was defined by matching to the ICD-8 or ICD-9 Swedish version diagnostic codes or related variables except for one item “incubator”, which cannot be matched to any specific ICD codes or variables in the data directly. The Lewis-Murray Scale defines item endorsement as “equivocal” when standards are diminished from “definite” criteria or there is a deficiency of information. For example, “definite” low birth weight is defined as birth weight lower than 2000g, whereas “equivocal” low birth weight is defined as birth weight lower than 2500g. Thus, more individuals were counted when defining low birth weight by including both “definite” and “equivocal” criteria. Additionally, the Lewis-Murray scale was used as either an ordinal or binary tool in this study. The categories were defined by the total number of ELCs for each person. The binary tool was defined by whether the individual had any ELCs or none.

**2. Maudsley Family Study of Psychosis (MFS)**

The Maudsley Family Study of Psychosis recruited participants from all over the UK through advertisements in the local press, by attendance to patient and carer group meetings hosted by voluntary sector organisations and through referrals from clinicians. Inclusion criteria for patients: Having a diagnosis of schizophrenia, schizoaffective disorder, bipolar disorder with a history of psychotic symptoms and other psychotic disorders. Any family members with or without a history of psychosis were also eligible. Controls were included if they had no personal or family history of psychosis.

To allow for a DSM-IV diagnosis to be ascertained or ruled out, all participants (including controls and unaffected family members) underwent a structured clinical interview with either the Schedule for Affective Disorders and Schizophrenia (SADS) or the Structured Clinical Interview for DSM Disorders (SCID).^3,4^

Genomic DNA was obtained from blood for all participants. Samples were sent for genotyping with the Genome-wide Human SNP Array 6.0 at Affymetrix Services Lab (<http://www.affymetrix.com>). Genotype calling was conducted using the CHIAMO algorithm^5^ modified for use with the Affymetrix 6.0 genotyping array. Standard quality control criteria were applied as described in previous papers.^6,7^

Information about Obstetric Complications (OCs) was collected interviewing the mother of patients, siblings, or controls using the Lewis-Murray scale.^2^ Participants provided written informed consent and the study was approved by the ethical committee at the Institute of Psychiatry.

**3. Verona**

Cases were obtained from the Psychosis Incident Cohort Outcome Study (PICOS), a multisite collaborative research on first episode psychosis (FEP) patients, residents in the Veneto Region, North-eastern Italy.^8,9^ Patients, aged 15-54 years, were included if they had had first contact with any mental health service in the PICOS area during the index period (Jan 1, 2005–Dec 31, 2007), with evidence of (a) at least one of the following symptoms: hallucinations, delusions, qualitative speech disorder, qualitative psychomotor disorder, bizarre or grossly inappropriate behaviour, or (b) at least two of the following symptoms: loss of interest, initiative and drive, social withdrawal, episodic severe excitement, purposeless destructiveness, overwhelming fear, marked self-neglect. The exclusion criteria were: (1) any previous presentation or treatment for psychotic illness for more than 3 months; (2) mental disorders due to a general medical condition; (3) moderate to severe mental retardation. Each FEP patient was approached and invited to undertake standardized assessments. The diagnosis was confirmed 6 months after inclusion into the study using the IGC (Item Group Checklist) of the SCAN, which allows one to rate information from case records, integrated with interviews with the patient case manager if needed. Only patients with a confirmed ICD-10 diagnosis of psychosis (F1x.4; F1x.5; F1x.7; F20–29; F30.2, F31.2, F31.5, F31.6, F32.3, F33.3) were included in the PICOS.

Controls were recruited through notices posted at the Verona University Hospital, Verona, Italy.^10^ Individuals presenting a history of neurological or psychiatric diseases, prior traumatic brain injury, or mental retardation (IQ<70) were excluded. The absence of psychiatric disorder was ascertained with the Mini International Neuropsychiatric Interview (M.I.N.I. Plus) and the Structured Clinical Interview for DSM disorders (SCID-II). In addition, being pregnant or in lactation represented an exclusion criterion.

Information about Obstetric Complications (OCs) was collected interviewing the mother of patients or controls using the Lewis-Murray scale.^2^ The scale rates 15 obstetric complications as absent or definitely present; 9 of the exposures can also be rated as equivocally present. As done previously, we considered either definite or equivocal exposure to any complication of pregnancy or labour as positive exposure.

Venous blood samples (15 ml) were collected in EDTA containing tubes from each participant (cases and controls), and DNA was extracted from blood leukocytes by using a commercial kit (ABgene, Blenheim Road, Epson, Surrey, UK).

Written informed consent was obtained from all participants, as approved by both the Ethics Committee of the Verona University Hospital and the local Ethics Committees of PICOS participating sites.

**4. UK Biobank**

In the present study we analysed data from the UK Biobank (UKBB) health resource. The recruitment process was coordinated around 22 centres in the UK.^11^ Individuals within travelling distance of these centres were identified using NHS patient registers (response rate = 5.47%).^12^ All participants provided written consent and the current study was ethically approved by the UK Biobank Ethics and Governance Council (REC reference 11/NW/0382; UK Biobank application reference 18177).

Genotype quality control (QC) procedures were primarily performed by the UK Biobank and are described elsewere.^13,14^ Blood samples from 500,000 UK Biobank (UKB) participants were genotyped using the UK BiLEVE array or the UK Biobank axiom array. Genotyped SNPs were removed if they had missingness < 0.02 and minor allele frequency (MAF) < 0.01. SNPs deviating from Hardy-Weinburg equilibrium (HWE) were removed at a threshold of P < 10^-08^. Participants were removed from the dataset if they had missingness < 0.01. A subgroup of European ancestry inferred individuals were defined using 4-means clustering applied to the first two principal components of the genotype data. One of each pair of related individuals was removed using a relatedness criterion (pi-hat) of < 0.088. Participants were removed if their self-reported and genotype inferred sex didn’t match. This QC process resulted in a data set of 544,633 SNPs and 385,793 samples available for analysis with genotype data.

The participants birth weight was obtained during a verbal interview with a trained staff member during the UKBB baseline assessment. A binary variable for ‘low birth weight’ was defined, based on participants who had a birth weight of less than 2.5 kg. To identify 326 participants with schizophrenia we used self-reported measures (self-report code 1989) obtained during the verbal interview and linked hospital episode statistics (ICD-9 2951 & 1956; ICD-10 F20). We generated polygenic risk scores (PRS) for the participants using publicly available GWAS summary statistics on schizophrenias.^15^ Polygenic risk scores were calculated using PRSice-2 software as the sum of disease-associated alleles, weighted by the effect size estimated in the discovery GWAS.^16^ PRS were calculated at three P-value thresholds: 5e-08, 1e-06 and 0.1. The association between the PRS and birth weight/status were estimated in a logistic regression adjusted for 6 UKBB derived ancestry informed principal components. Effects reported are per a standard deviation change in PRS.

**5. Cardiff**

Study individuals came from the CardiffCOGS (COGnition in Schizophrenia) sample, which has been previously described.^17,18^ CardiffCOGS is a sample of patients with clinically diagnosed schizophrenia or related psychotic disorder recruited from secondary care NHS mental health services in Wales and England. All participants completed a SCAN interview and had a case note review followed by consensus research diagnostic procedures. Participants were included in this study if they had a DSM-IV diagnosis of schizophrenia or schizoaffective disorder depressed type. As part of the study interview, participants were asked whether they had experienced birth and/or pregnancy complications and gave descriptive details of these complications. Trained researchers subsequently reviewed participant psychiatric medical records to validate participant self-report of obstetric complications.

Genotyping and calculation of polygenic risk scores for schizophrenia has been fully described elsewhere.^18^ Briefly, the CardiffCOGS sample was genotyped on either the Illumina HumanOmniExpressExome-8 or the Illumina HumanOmniExpress-12 array. After standard quality control procedures, imputation was performed using IMPUTE2 and the 1000 Genomes and UK10K reference panels. Polygenic risk scores for schizophrenia were calculated based on the largest published schizophrenia genome-wide association study^19^ and following the method described by Wray et al.^20^

**Supplementary References**

1 Ripke, S. et al. Genome-wide association analysis identifies 13 new risk loci for schizophrenia. Nature Genetics **45**, 1150-+, doi:10.1038/ng.2742 (2013).

2 Lewis, S. W., Owen, M. J. & Murray, R. M. in Schizophrenia: A Scientific Focus (eds S.C. Schulz & C.A. Tamminga) 56-68 (Oxford University Press, 1989).

3 Endicott, J. & Spitzer, R. L. A diagnostic interview: the schedule for affective disorders and schizophrenia. Arch Gen Psychiatry **35**, 837-844 (1978).

4 Spitzer, R. L., Williams, J. B. W., Gibbon, M. & First, M. B. The Structured Clinical Interview for Dsm-III-R (Scid) .1. History, Rationale, and Description. Archives of General Psychiatry **49**, 624-629 (1992).

5 Marchini, J., Howie, B., Myers, S., McVean, G. & Donnelly, P. A new multipoint method for genome-wide association studies by imputation of genotypes. Nat Genet **39**, 906-913, doi:10.1038/ng2088 (2007).

6 Bramon, E. et al. A genome-wide association analysis of a broad psychosis phenotype identifies three loci for further investigation. Biol Psychiatry **75**, 386-397, doi:10.1016/j.biopsych.2013.03.033 (2014).

7 Calafato, M. S. et al. Use of schizophrenia and bipolar disorder polygenic risk scores to identify psychotic disorders. Br J Psychiatry **213**, 535-541, doi:10.1192/bjp.2018.89 (2018).

8 Lasalvia, A. et al. Psychosis Incident Cohort Outcome Study (PICOS). A multisite study of clinical, social and biological characteristics, patterns of care and predictors of outcome in first-episode psychosis. Background, methodology and overview of the patient sample. Epidemiol Psychiatr Sci **21**, 281-303, doi:10.1017/S2045796012000315 (2012).

9 Ira, E. et al. Positive symptoms in first-episode psychosis patients experiencing low maternal care and stressful life events: a pilot study to explore the role of the COMT gene. Stress **17**, 410-415, doi:10.3109/10253890.2014.948841 (2014).

10 Lopizzo, N. et al. Transcriptomic analyses and leukocyte telomere length measurement in subjects exposed to severe recent stressful life events. Transl Psychiatry **7**, e1042, doi:10.1038/tp.2017.5 (2017).

11 Allen, N. E., Sudlow, C., Peakman, T., Collins, R. & Biobank, U. K. UK biobank data: come and get it. Sci Transl Med **6**, 224ed224, doi:10.1126/scitranslmed.3008601 (2014).

12 Sudlow, C. et al. UK biobank: an open access resource for identifying the causes of a wide range of complex diseases of middle and old age. PLoS Med **12**, e1001779, doi:10.1371/journal.pmed.1001779 (2015).

13 Bycroft, C. et al. The UK Biobank resource with deep phenotyping and genomic data. Nature **562**, 203-209, doi:10.1038/s41586-018-0579-z (2018).

14 Coleman, J. R. I. et al. Genome-wide gene-environment analyses of major depressive disorder and reported lifetime traumatic experiences in UK Biobank. bioRxiv, 247353, doi:10.1101/247353 (2019).

15 Schizophrenia Working Group of the Psychiatric Genomics Consortium. Biological insights from 108 schizophrenia-associated genetic loci. Nature **511**, 421-427, doi:10.1038/nature13595 (2014).

16 Choi, S. W. & O'Reilly, P. F. PRSice-2: Polygenic Risk Score software for biobank-scale data. GigaScience **8**, doi:10.1093/gigascience/giz082 (2019).

17 Lynham, A. J. et al. Examining cognition across the bipolar/schizophrenia diagnostic spectrum. J Psychiatry Neurosci **43**, 245-253 (2018).

18 Legge, S. E. et al. Clinical indicators of treatment-resistant psychosis. Br J Psychiatry, 1-8, doi:10.1192/bjp.2019.120 (2019).

19 Pardinas, A. F. et al. Common schizophrenia alleles are enriched in mutation-intolerant genes and in regions under strong background selection. Nat Genet **50**, 381-389, doi:10.1038/s41588-018-0059-2 (2018).

20 Wray, N. R. et al. Research review: Polygenic methods and their application to psychiatric traits. J Child Psychol Psychiatry **55**, 1068-1087, doi:10.1111/jcpp.12295 (2014).

**Supplementary Figure 1. Meta-analysis of associations between PRS and OCs in cases**

**A. PRS at P_T_ < 5x10^-8^**

**
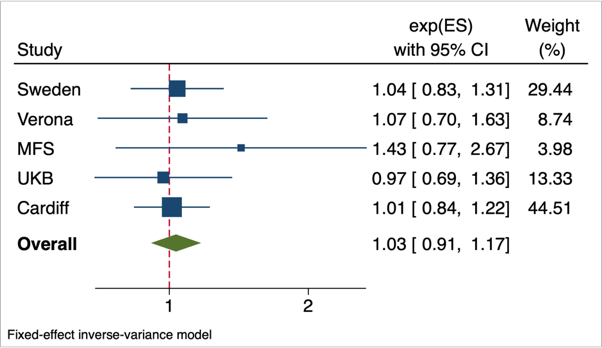
**

**B. PRS at P_T_ < 1x10^-6^**

**
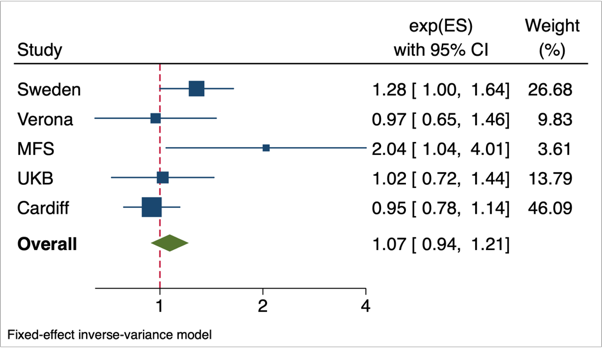
**

**Supplementary Figure 2. Meta-analysis of associations between PRS and case-control status stratified by the presence or absence of OCs**

**A. PRS at P_T_ < 5x10^-8^**

**
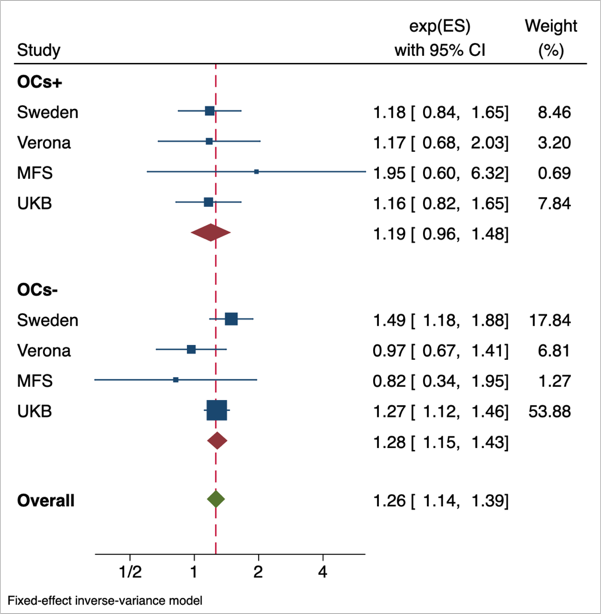
**

**B. PRS at P_T_ < 1x10^-6^**

**
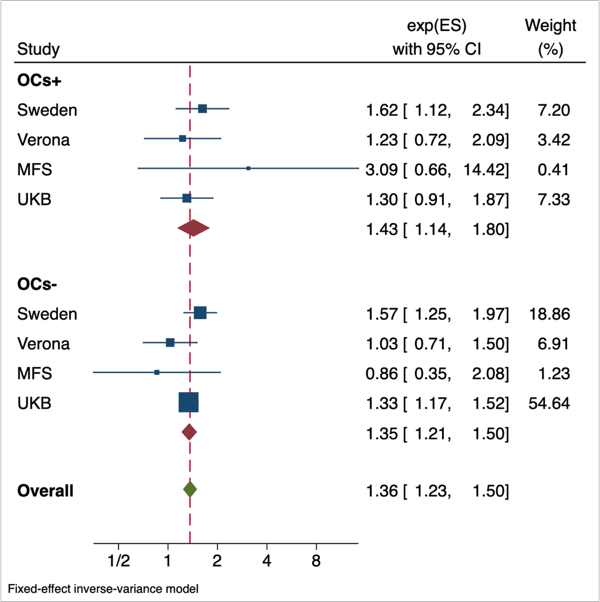
**

**Supplementary Table 1. Associations between OCs, case-control status, and PRS including SNPs at P_T_ < 0.1**

| **Sample** | **PRS & OCs** | | | **PRS & status** | | | | **PRS by OCs** | |
| --- | --- | --- | --- | --- | --- | --- | --- | --- | --- |
|  |  | exp(B) | P |  | exp(B) | R^2^ | P | exp(B) | P |
| Sweden | All | 0.90 | 0.36 | All | 2.87 | 0.157 | **8.4e-15** | 1.16 | 0.61 |
|  | Controls | 0.85 | 0.30 | OCs- | 2.68 | 0.145 | **2.0e-11** |  |  |
|  | Cases | 0.97 | 0.85 | OCs+ | 3.45 | 0.178 | **8.7e-08** |  |  |
| Verona | All | 0.95 | 0.78 | All | 1.62 | 0.038 | **0.007** | 1.16 | 0.73 |
|  | Controls | 0.96 | 0.88 | OCs- | 1.65 | 0.033 | **0.038** |  |  |
|  | Cases | 0.97 | 0.90 | OCs+ | 1.64 | 0.037 | 0.11 |  |  |
| MFS | All | 1.79 | **0.01** | All | 2.89 | 0.162 | **0.005** | 3.27 | 0.29 |
|  | Controls | 1.07 | 0.90 | OCs- | 2.15 | 0.103 | 0.06 |  |  |
|  | Relatives | 2.33 | 0.10 | OCs+ | 8.70 | 0.294 | 0.05 |  |  |
|  | Cases | 2.84 | **0.01** |  |  |  |  |  |  |
| UKB | All | 1.009 | 0.24 | All | 1.81 | 0.022 | **8.8e-25** | 0.77 | 0.14 |
|  | Controls | 1.009 | 0.22 | OCs- | 1.87 | 0.024 | **2.9e-24** |  |  |
|  | Cases | 0.82 | 0.29 | OCs+ | 1.43 | 0.008 | **0.034** |  |  |
| Cardiff | Cases | 1.11 | 0.46 |  |  |  |  |  |  |

OCs: obstetric complications; PRS: schizophrenia polygenic risk score adjusted for 10 PCs

PRS & OCs: Association between PRS and history of OCs in the total sample (All), controls, cases (and relatives in the MFS) separately

PRS & status: Association between PRS and case-control status in the total sample (All), the subsample without OCs history (OCs-), and the subsample with OCs history (OCs+)

PRS by OCs: Coefficient and p-value of the Interaction term PRS*OC

P-values < 0.05 in bold

**Supplementary Table 2. Association with case-control status in the Swedish cohort of pathways enriched for placental genes at P_T_ < 5x10^-8^**

| FULL_NAME | N Genes | BETA | BETA  (STD) | SE | P |
| --- | --- | --- | --- | --- | --- |
| UNFOLDED_PROTEIN_RESPONSE | 5 | -5.35 | -0.46 | 4.14 | 0.90 |
| MITOCHONDRIAL_DYSFUNCTION | 7 | 0.50 | 0.05 | 1.33 | 0.35 |
| FCG_RECEPTOR-MEDIATED_PHAGOCYTOSIS_IN_MACROPHAGES_AND_MONOCYTES | 4 | 3.66 | 0.28 | 4.35 | 0.20 |
| ALDOSTERONE_SIGNALING_IN_EPITHELIAL_CELLS | 6 | 2.49 | 0.23 | 3.19 | 0.22 |
| PROTEIN_UBIQUITINATION_PATHWAY | 7 | 0.58 | 0.06 | 2.40 | 0.41 |
| FLT3_SIGNALING_IN_HEMATOPOIETIC_PROGENITOR_CELLS | 3 | -11.9 | -0.79 | 8.62 | 0.92 |
| GNRH_SIGNALING | 5 | -1.54 | -0.13 | 3.74 | 0.66 |

**Supplementary Table 3. Association with case-control status in the Swedish cohort of pathways enriched for placental genes at P_T_ < 10^-6^**

| FULL_NAME | N Genes | BETA | BETA (STD) | SE | P |
| --- | --- | --- | --- | --- | --- |
| ANGIOPOIETIN_SIGNALING | 7 | -3.56 | -0.27 | 4.44 | 0.79 |
| LPS-STIMULATED_MAPK_SIGNALING | 7 | -3.62 | -0.27 | 4.92 | 0.77 |
| FLT3_SIGNALING_IN_HEMATOPOIETIC_PROGENITOR_CELLS | 6 | -16.03 | -1.11 | 7.62 | 0.98 |
| MITOCHONDRIAL_DYSFUNCTION | 10 | 0.79 | 0.07 | 1.43 | 0.29 |
| UNFOLDED_PROTEIN_RESPONSE | 6 | -4.27 | -0.30 | 4.01 | 0.86 |
| EPHRIN_RECEPTOR_SIGNALING | 10 | 1.23 | 0.11 | 4.25 | 0.39 |
| IL-6_SIGNALING | 8 | -2.84 | -0.23 | 3.53 | 0.79 |
| GNRH_SIGNALING | 8 | -4.28 | -0.34 | 3.66 | 0.88 |
| RENAL_CELL_CARCINOMA_SIGNALING | 6 | -2.61 | -0.18 | 5.24 | 0.69 |
| HIF1A_SIGNALING | 7 | -3.46 | -0.26 | 5.12 | 0.75 |
| B_CELL_RECEPTOR_SIGNALING | 8 | -6.85 | -0.55 | 4.23 | 0.95 |
| ENDOMETRIAL_CANCER_SIGNALING | 5 | -1.07 | -0.07 | 4.48 | 0.59 |
| ERK/MAPK_SIGNALING | 9 | -3.31 | -0.28 | 4.65 | 0.76 |
| PROSTATE_CANCER_SIGNALING | 6 | -9.74 | -0.67 | 6.47 | 0.93 |
| ERBB_SIGNALING | 6 | -1.46 | -0.10 | 4.43 | 0.63 |
| INSULIN_RECEPTOR_SIGNALING | 6 | 1.09 | 0.08 | 6.32 | 0.43 |
| HYPOXIA_SIGNALING_IN_THE_CARDIOVASCULAR_SYSTEM | 5 | -9.79 | -0.62 | 6.43 | 0.93 |
| MOLECULAR_MECHANISMS_OF_CANCER | 12 | -2.45 | -0.24 | 2.65 | 0.82 |
| NRF2-MEDIATED_OXIDATIVE_STRESS_RESPONSE | 8 | -1.71 | -0.14 | 2.67 | 0.74 |
| UVC-INDUCED_MAPK_SIGNALING | 4 | -2.83 | -0.16 | 5.64 | 0.69 |
| IL-3_SIGNALING | 4 | 1.55 | 0.09 | 5.78 | 0.39 |
| PEDF_SIGNALING | 5 | -17.37 | -1.10 | 8.49 | 0.98 |
| GLUCOCORTICOID_RECEPTOR_SIGNALING | 10 | -2.40 | -0.21 | 2.17 | 0.86 |
| ROLE_OF_RIG1-LIKE_RECEPTORS_IN_ANTIVIRAL_INNATE_IMMUNITY | 4 | -3.53 | -0.20 | 5.19 | 0.75 |
| NATURAL_KILLER_CELL_SIGNALING | 5 | 1.18 | 0.07 | 4.52 | 0.40 |
| CORTICOTROPIN_RELEASING_HORMONE_SIGNALING | 6 | -4.34 | -0.30 | 4.77 | 0.82 |
| VEGF_FAMILY_LIGAND-RECEPTOR_INTERACTIONS | 5 | 1.86 | 0.12 | 5.72 | 0.37 |
| ACUTE_MYELOID_LEUKEMIA_SIGNALING | 5 | -7.04 | -0.45 | 9.48 | 0.77 |
| ROLE_OF_MACROPHAGES_FIBROBLASTS_AND_ENDOTHELIAL_CELLS_IN_RHEUMATOID_ARTHRITIS | 10 | -3.03 | -0.27 | 4.16 | 0.77 |
| SYNAPTIC_LONG_TERM_POTENTIATION | 6 | -1.78 | -0.12 | 4.88 | 0.64 |
| PROTEIN_UBIQUITINATION_PATHWAY | 9 | 0.39 | 0.03 | 2.37 | 0.44 |
| PI3K/AKT_SIGNALING | 5 | -1.82 | -0.12 | 3.64 | 0.69 |
| UVB-INDUCED_MAPK_SIGNALING | 4 | -2.38 | -0.13 | 5.61 | 0.66 |
| NEUREGULIN_SIGNALING | 5 | 1.76 | 0.11 | 1.69 | 0.15 |
| APOPTOSIS_SIGNALING | 5 | -1.82 | -0.12 | 3.64 | 0.69 |
| VEGF_SIGNALING | 5 | 0.42 | 0.03 | 13.85 | 0.49 |
| FCG_RECEPTOR-MEDIATED_PHAGOCYTOSIS_IN_MACROPHAGES_AND_MONOCYTES | 4 | 3.46 | 0.20 | 4.46 | 0.22 |
| ATM_SIGNALING | 4 | -14.49 | -0.82 | 6.29 | 0.99 |
| AXONAL_GUIDANCE_SIGNALING | 12 | 1.79 | 0.17 | 2.38 | 0.23 |
| ERBB4_SIGNALING | 4 | -0.10 | -0.01 | 3.37 | 0.51 |
| 4-1BB_SIGNALING_IN_T_LYMPHOCYTES | 3 | -18.95 | -0.93 | 8.83 | 0.98 |
| PROTEIN_KINASE_A_SIGNALING | 11 | 0.43 | 0.04 | 2.41 | 0.43 |
| IL-8_SIGNALING | 7 | 0.24 | 0.02 | 4.27 | 0.48 |
| CDK5_SIGNALING | 5 | -4.82 | -0.30 | 5.51 | 0.81 |
| ILK_SIGNALING | 7 | -5.04 | -0.38 | 7.45 | 0.75 |
| ERK5_SIGNALING | 4 | -9.22 | -0.52 | 8.46 | 0.86 |
| ESTROGEN-DEPENDENT_BREAST_CANCER_SIGNALING | 4 | -10.04 | -0.57 | 8.54 | 0.88 |
| ROLE_OF_PI3K/AKT_SIGNALING_IN_THE_PATHOGENESIS_OF_INFLUENZA | 4 | -4.70 | -0.27 | 5.82 | 0.79 |
| NITRIC_OXIDE_SIGNALING_IN_THE_CARDIOVASCULAR_SYSTEM | 5 | 2.46 | 0.16 | 5.51 | 0.33 |
| MIF-MEDIATED_GLUCOCORTICOID_REGULATION | 3 | -7.29 | -0.36 | 9.79 | 0.77 |
| 3-PHOSPHOINOSITIDE_DEGRADATION | 5 | 3.45 | 0.22 | 2.48 | 0.08 |
| RAC_SIGNALING | 5 | -9.90 | -0.63 | 9.25 | 0.86 |
| SUPERPATHWAY_OF_INOSITOL_PHOSPHATE_COMPOUNDS | 6 | 3.38 | 0.23 | 2.47 | 0.09 |
| CELL_CYCLE_REGULATION_BY_BTG_FAMILY_PROTEINS | 3 | -2.12 | -0.10 | 2.00 | 0.86 |
| IL-17A_SIGNALING_IN_FIBROBLASTS | 3 | -18.95 | -0.93 | 8.83 | 0.98 |
| ERYTHROPOIETIN_SIGNALING | 4 | -1.37 | -0.08 | 5.12 | 0.61 |
| NEUROTROPHIN/TRK_SIGNALING | 4 | -10.04 | -0.57 | 8.54 | 0.88 |
| REMODELING_OF_EPITHELIAL_ADHERENS_JUNCTIONS | 4 | -1.22 | -0.07 | 5.05 | 0.60 |
| NGF_SIGNALING | 5 | -1.89 | -0.12 | 4.92 | 0.65 |
| GROWTH_HORMONE_SIGNALING | 4 | 0.85 | 0.05 | 5.29 | 0.44 |
| ROLE_OF_MAPK_SIGNALING_IN_THE_PATHOGENESIS_OF_INFLUENZA | 4 | -34.51 | -1.95 | 15.12 | 0.99 |
| FC_EPSILON_RI_SIGNALING | 4 | -2.83 | -0.16 | 5.64 | 0.69 |
| FMLP_SIGNALING_IN_NEUTROPHILS | 5 | -0.94 | -0.06 | 4.95 | 0.57 |
| ALDOSTERONE_SIGNALING_IN_EPITHELIAL_CELLS | 6 | 2.80 | 0.19 | 3.27 | 0.20 |
| RENIN-ANGIOTENSIN_SIGNALING | 5 | -5.55 | -0.35 | 5.52 | 0.84 |
| COMPLEMENT_SYSTEM | 3 | -6.83 | -0.33 | 3.50 | 0.97 |
| ROLE_OF_TISSUE_FACTOR_IN_CANCER | 5 | -4.61 | -0.29 | 5.52 | 0.80 |
